# Factors Related to the Care Management Practice for Old Public Assistance Recipients in Osaka City of Japan

**DOI:** 10.1093/geroni/igab046.3394

**Published:** 2021-12-17

**Authors:** Takako Ayabe, Yoshihito Takemoto, Shinichi OKADA, Johannes Kiener, Masakazu SHIRASAWA

**Affiliations:** 1 BAIKA Women’s university, ibaraki, Osaka, Japan; 2 Okayama Prefectural University, Soja, Okayama, Japan; 3 Osaka City University, Osaka, Osaka, Japan; 4 Saitama University, Saitama, Saitama, Japan; 5 International University of Health and Welfare, Minato City, Tokyo, Japan

## Abstract

The research was conducted between January 22 and February 25, 2021. The data was collected by self-administered questionnaires mailed to the participants at 800 care management centers and comprehensive community support centers in Osaka City. The centers were randomly selected. The response rate was 19.1%. The independent variables were: obtaining the qualification of a Senior Care Manager (SCM), who was a qualified person that acquired advanced knowledge and skills in care management by advanced training; experience years in Social Work (SW); experience years in care management; experiences in training programs for team approach; and experiences in training programs for supporting Old Public Assistance Recipients (OPAR). The dependent variables were the categorized contents in the Care Management Practice for old public assistance recipients. They were: Care planning and Implementation (CI); Assessment; Financial Support and Evaluation (FSE); Contract and Explanations in care management; Coordinating Informal support and Formal services in Care planning; and Arrangements in Financial supports for Formal service costs. The Structural Equation Modeling was performed for the examinations of the relationships. As a result, the goodness of the fit indices was acceptable, and we retained the models. In correlational analyses, CI and Assessment were significantly correlated with SCM (p<.05). FSE was significantly correlated with SW (p<.001) and OPAR (p<.05). In conclusion, the results implied that advanced qualification of a Senior Care Manager and a specified training program for supporting old public assistance recipients were effective in providing appropriate care management services.

